# Spatial variations in urban park accessibility and equity under multimodal travel: a case study of Beijing

**DOI:** 10.3389/fpubh.2026.1953457

**Published:** 2026-10-09

**Authors:** Sisi Song, Haiyuan Quan, Lijian Ren, Hengyi Zang, Zening Li, Xiaodie Liu

**Affiliations:** 1 School of Architecture, Tianjin University, Tianjin, China; 2 School of Architecture and Art, North China University of Technology, Beijing, China

**Keywords:** multimodal accessibility, park green spaces, spatial equity, three-step floating catchment area method, urban fringe

## Abstract

**Introduction:**

Equity in urban green space depends not only on whether aggregate park supply is sufficient, but also on how supply aligns with population demand and everyday travel conditions.

**Methods:**

Taking Beijing as a case study, we represented supply by park area and demand by population distribution. We used the three-step floating catchment area method (3SFCA) to assess accessibility to park green spaces under walking, cycling, and driving. Population-weighted Lorenz curves and Gini coefficients, local bivariate Moran's I, and importance-performance analysis (IPA) were then used to identify inequities and priority areas.

**Results:**

Accessibility and equity exhibited marked spatial differences. The urban core had the lowest mean accessibility and the strongest overlap between high population demand and low accessibility, making it the primary area for improvement. The urban fringe had higher overall accessibility, but inadequate local green-space service remained pronounced under walking. This pattern reflected a transitional inequity between residential expansion and park provision. The suburb had high mean accessibility but an unequal distribution of walking opportunities, indicating that large-area green-space supply does not necessarily translate into daily access for residents of suburban towns. Park-size classes also performed different functions. District green spaces formed the most stable backbone of area-based accessibility. Regional green spaces relied more on cycling and driving for cross-zone service. Small and micro green spaces and community green spaces were critical for closing local-service gaps in high-density areas.

**Discussion:**

Beijing should therefore shift from simply increasing green-space area toward coordinated zone-, class-, and mode-specific provision. Local green spaces should be prioritized in the urban core. The urban fringe requires stronger active-travel connections between expanding residential areas and district green spaces. In the suburb, daily access should be improved while large green spaces are protected.

## 1 Introduction

Urban park green spaces support recreation and routine physical activity and contribute to psychological restoration, social interaction, and environmental health, making them important public health resources (1, 2). Green-space equity therefore cannot be judged solely by total urban park area or per-capita green space. Even under equivalent supply, usable opportunities decline when parks are distant from population concentrations or their entrances are difficult to reach. Opportunities also decline when parks lie beyond residents' routine activity spaces (3, 4). Small and micro green spaces embedded in high-demand communities may have limited area but can support more frequent daily use. Previous studies have also identified systematic differences in green-space exposure and opportunities to access parks among urban areas and social groups (5–8). Green-space equity assessment should consequently shift from asking how much green space a city contains to asking whether residents can reach suitable parks at acceptable travel costs. Such an assessment must jointly consider supply capacity, population competition, and travel impedance.

Pronounced spatial gradients created by urbanization make park accessibility and equity especially complex in megacities (9–11). A comparison of Beijing's urban core, urban fringe, and peripheral rural areas identified a transitional disadvantage in employment and public-service accessibility in the urban fringe (12). Studies of park distribution and environmental justice have revealed supply pressure in the central city and unequal opportunities among population groups (13–15). Research on urban expansion has shown that newly added green space does not necessarily improve population access at the same pace (16, 17). Studies using multi-source geographic and mobile-phone data further indicate that realized park service areas vary with park characteristics, travel modes, and user groups (18–20). Together, this evidence indicates that park equity in megacities should be examined along the continuous spatial gradient formed by the urban core, urban fringe, and suburb.

Recent people-centered assessments have used mobile phone signaling data to estimate realized visitation and temporally varying demand, revealing supply-demand mismatches that static residential-population indicators may not capture (21, 22). These dynamic approaches shift the emphasis from potential residential opportunity to observed park use. The present study instead focuses on residential equity in potential accessibility, for which the spatial distribution of the resident population provides the demand base.

Research on park equity has expanded from geometric proximity to supply-demand competition and social disparities, yet analytical scales and indicator definitions remain highly inconsistent (23). Evidence for Beijing still focuses mainly on the central city, a single administrative scale, or particular population groups (24–26). Recent studies have incorporated climate resilience and perceived quality (27, 28), but interactions among the urban core, urban fringe, and suburb remain insufficiently examined. These zones differ substantially in population demand intensity and park-size structure. However, whether different park-size classes provide differentiated services through walking, cycling, and driving has not been compared within a unified framework. Ignoring interactions among urban spatial gradients, park-size classes, and travel modes may lead the aggregate advantage created by large suburban parks to be misinterpreted as equitable daily service.

Floating catchment area methods provide a unified framework for integrating facility supply, population demand, and travel impedance (29, 30). Extensions of the two-step floating catchment area method introduced distance decay and variable catchment sizes, reducing bias associated with binary threshold assumptions (31–33). The three-step floating catchment area method further incorporates demand-point choice weights among candidate facilities and thus captures both facility attractiveness and demand competition (34). In park green-space research, floating catchment frameworks have incorporated park size, service hierarchy, and multiple travel modes (35–37) and have been extended to multimodal 3SFCA equity assessment (38). These developments support comparisons among urban zones and park-size classes but have not fully explained differentiated service structures along Beijing's urban-rural gradient.

Mean accessibility alone cannot determine whether service opportunities are equitably distributed among residents or whether high population demand forms significant spatial associations with low accessibility. Population-weighted Lorenz curves and Gini coefficients measure the concentration of accessible opportunities across the population (39). Local bivariate Moran's I identifies local associations between one variable and the spatial lag of another (40, 41). Importance-performance analysis (IPA) combines demand and performance to classify planning units into four policy-relevant categories (42). This study sequentially assessed overall levels, population distribution, significant spatial clustering, and planning priorities. These components form a complementary diagnostic chain for evaluating equity.

To address these gaps, this study integrated data on park green spaces, population, walking, cycling, and driving networks, and urban zones in Beijing. Park area represented supply, and park green spaces were divided into small and micro, community, district, and regional classes. Three questions guided the study: (1) How do green-space supply structure, population demand intensity, and multimodal accessibility differ among the urban core, urban fringe, and suburb? (2) What service roles do the four park-size classes perform under walking, cycling, and driving? (3) Where are the Priority areas and significant spatial clusters characterized by high population and low accessibility, and how can these patterns inform zone- and class-specific optimization strategies? Figure 1 presents the analytical workflow.

**Figure 1:**
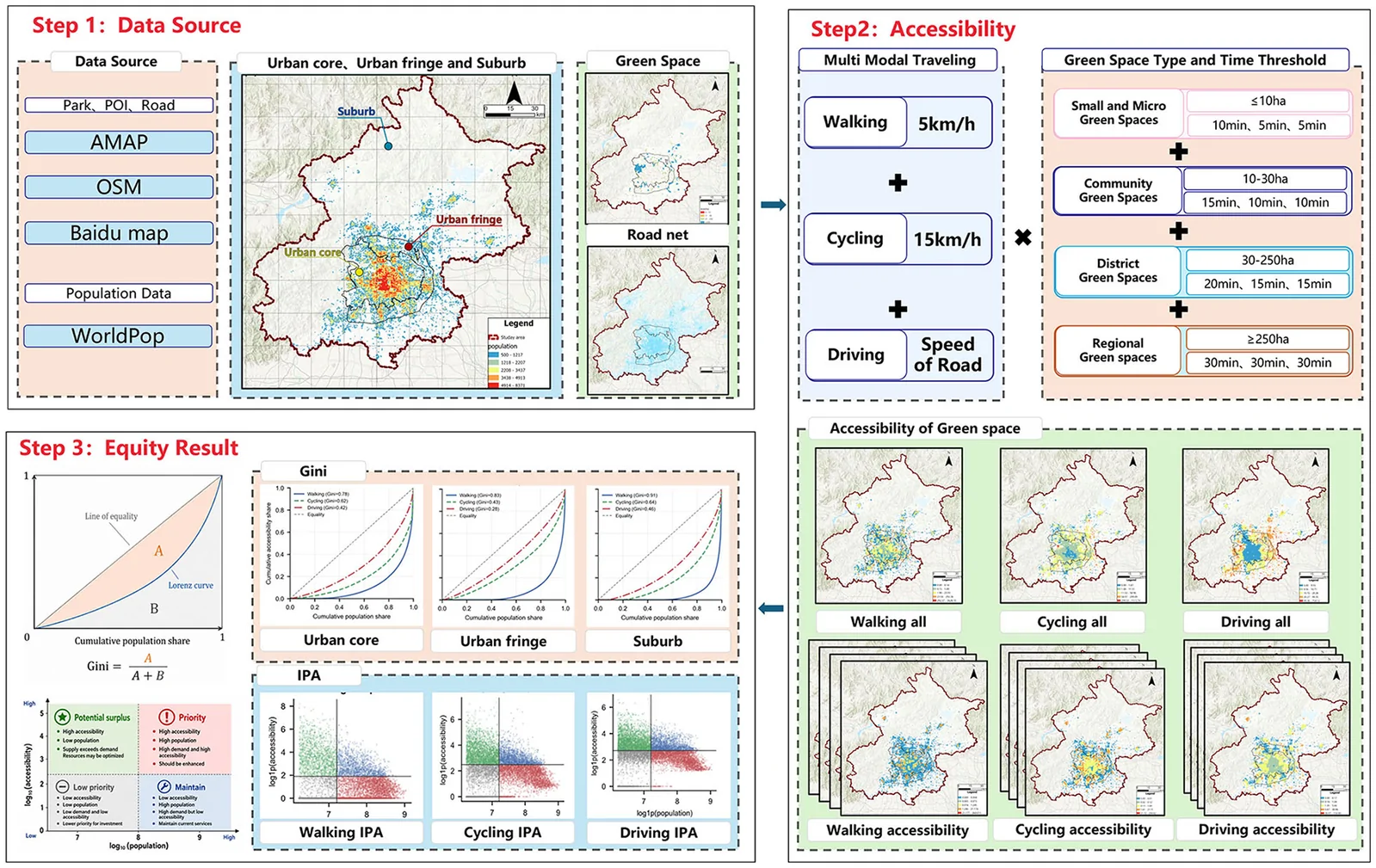
Analytical workflow. “Basemap: Esri World Topographic Map (ArcGIS Online), used under Esri's Terms of Use for Static Maps. Copyright © 2026 Esri and its licensors. All rights reserved.” The service-layer credits shown for the maps include Esri, © OpenStreetMap contributors, TomTom, Garmin, FAO, METI/NASA, USGS, NOAA, and CGIAR.

Accordingly, this study's contribution lies in applying a consistent multimodal supply-demand framework to diagnose how Beijing's urban core, urban fringe, and suburb convert four park-size classes into walking, cycling, and driving opportunities. This zone-by-class-by-mode design distinguishes the aggregate area advantage of large peripheral parks from daily walkable service and links the accessibility diagnosis to spatially explicit intervention priorities.

## 2 Materials and methods

### 2.1 Study area and data sources

Beijing was selected as the study area. It is located in the northern part of the North China Plain and covers approximately 16,400 km^2^. Beijing comprises 16 districts and has more than 20 million permanent residents. Rapid urbanization over recent decades has extended the built-up area toward the periphery. This process has produced a spatial structure linking a high-density urban core, a rapidly developing urban fringe, and a low-density periphery. Land use, population concentration, transport infrastructure, and public-service provision differ markedly among these zones.

The zoning scheme followed Zhao's comparative framework of Beijing's urban core, urban fringe, and peripheral rural areas and was spatially implemented using the zonal data adopted in this study (12) and *The Master Plan of Beijing (2016–2035)*. The urban core comprised Dongcheng, Xicheng, Haidian, Chaoyang, Fengtai, and Shijingshan districts. The urban fringe comprised areas within the Sixth Ring Road that were outside these six districts, and the remaining areas were classified as the suburb. This delineation was designed to represent differences in supply-demand conditions and travel environments among the high-density built-up area, the transitional expansion zone, and the low-density periphery. Figure 2 shows the study area and zonal boundaries.

**Figure 2:**
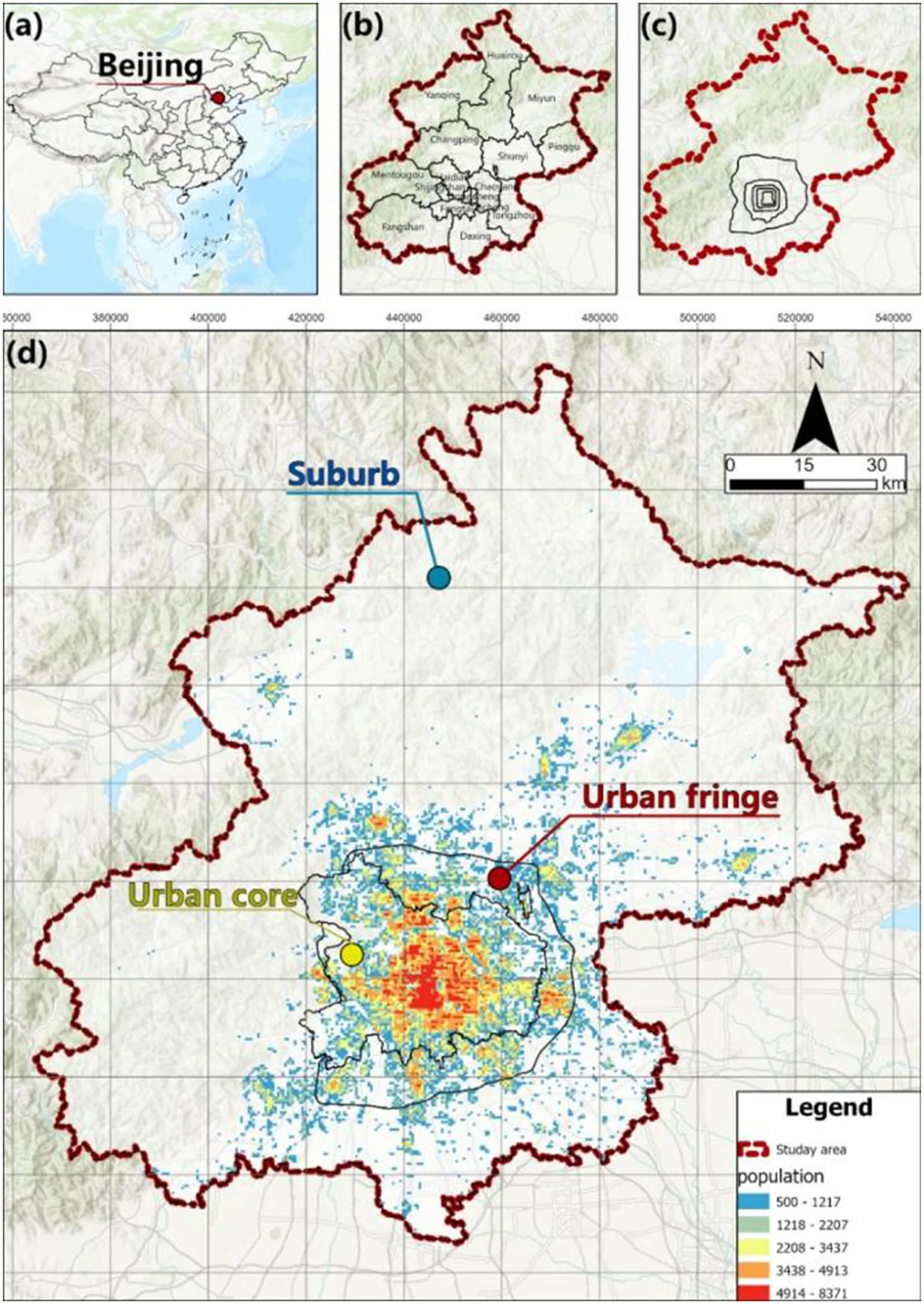
Study area and delineation of the urban core, urban fringe, and suburb. **(a)** Location of Beijing in China. **(b)** Administrative divisions of Beijing. **(c)** Ring roads in Beijing. **(d)** Delineation of the urban core, urban fringe, and suburb and the population distribution. “Basemap: Esri World Topographic Map (ArcGIS Online), used under Esri's Terms of Use for Static Maps. Copyright © 2026 Esri and its licensors. All rights reserved.” The service-layer credits shown for the maps include Esri, © OpenStreetMap contributors, TomTom, Garmin, FAO, METI/NASA, USGS, NOAA, and CGIAR.

Administrative boundaries, park green-space polygons, and points of interest were obtained from the Amap Open Platform. Road data were obtained from the Baidu Maps Open Platform and OpenStreetMap. All source datasets were from 2024. The park dataset integrated park vectors and points of interest from Amap with OpenStreetMap data. It contained 1,173 park green spaces with a total area of 60,292.6 ha (Figure 3).

**Figure 3:**
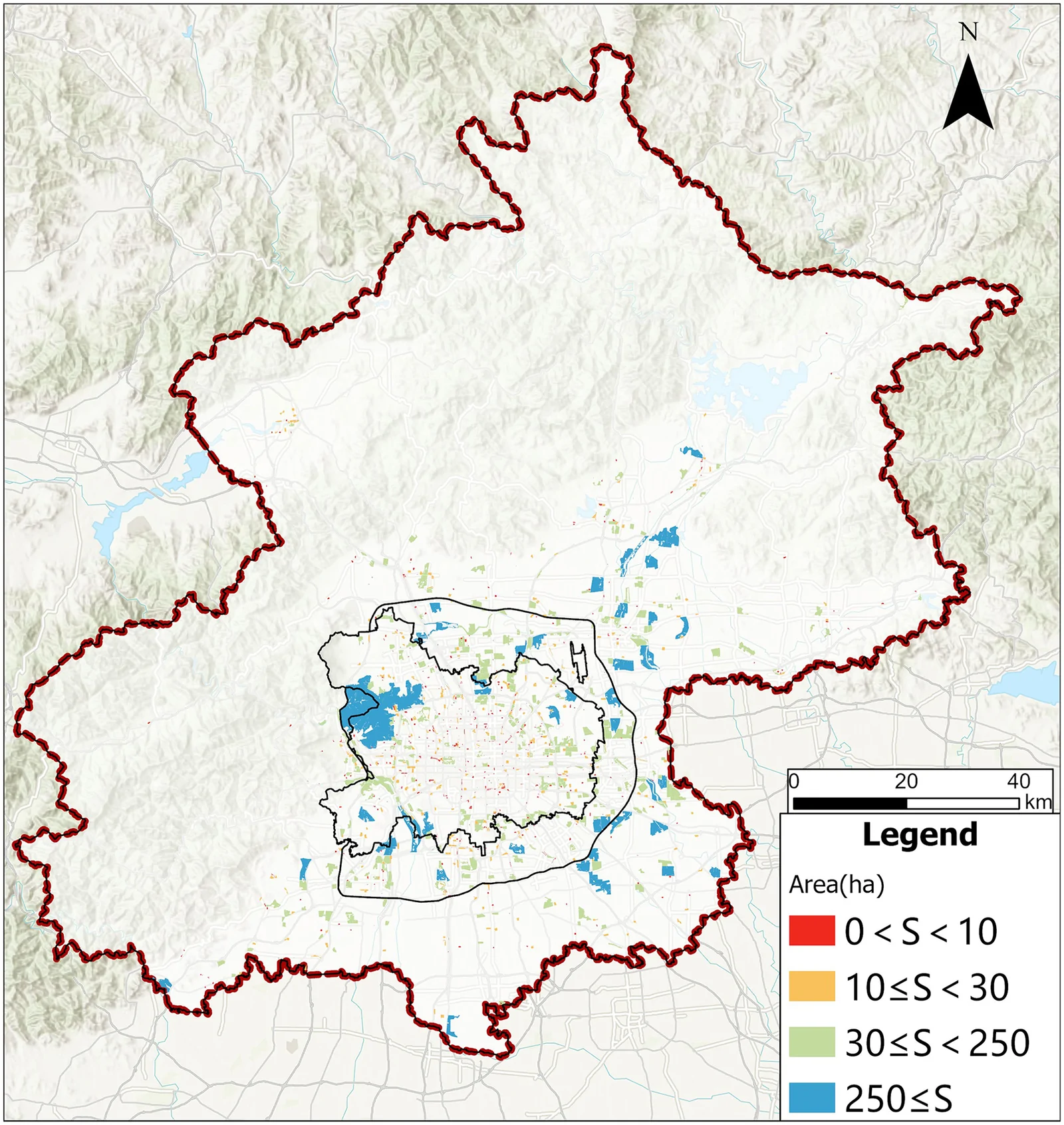
Spatial distribution of park green spaces in Beijing. “Basemap: Esri World Topographic Map (ArcGIS Online), used under Esri's Terms of Use for Static Maps. Copyright © 2026 Esri and its licensors. All rights reserved.” The service-layer credits shown for the maps include Esri, © OpenStreetMap contributors, TomTom, Garmin, FAO, METI/NASA, USGS, NOAA, and CGIAR.

The 2024 WorldPop population raster for China at 100 m resolution was aggregated to a 500 m × 500 m fishnet grid (43). The primary specification retained 10,088 grids with at least 500 residents as focal analytical units for consistency with the original study design and to focus downstream IPA and LISA classifications on more continuously settled residential cells. We treat 500 residents as an operational focal-sample rule rather than an imputation rule or a universal population-density standard. To evaluate its influence, two additional specifications were calculated: a demand-inclusive scenario in which all 40,390 grids with an estimated population greater than zero entered the 3SFCA competitive-demand denominator while the original focal evaluation set was retained, and a strict all-positive scenario in which all populated grids were used both as demand and evaluation units. This design separates the effect of low-density residents on park-level competition from the effect of changing the downstream evaluation sample. Data processing, calculation, and visualization were performed using GIS Pro 3.0.

All spatial data were projected to EPSG:32650. Walking, cycling, and driving accessibility were calculated on their corresponding road networks to reduce measurement bias caused by replacing actual travel paths with Euclidean distance (4). Because a citywide dataset of verified public park entrances was unavailable, intersections between the road network and park boundaries were used as network access points. When no intersection was identified, the nearest point between the park boundary and the road network was used as a substitute access point, and reaching this point was treated as entering the park. These access points should be interpreted as planning-scale proxies used to ensure consistent network-accessibility calculations, rather than as a complete inventory of public entrances verified through official records or field surveys.

### 2.2 Park-size classes and travel-time thresholds

Following previous research, park green spaces were divided into four size classes. These were small and micro green spaces (0 ≤ S < 10 ha), community green spaces (10 ≤ S < 30 ha), district green spaces (30 ≤ S < 250 ha), and regional green spaces (S ≥250 ha). Park size, service hierarchy, and travel mode jointly affect potential service areas and facility attractiveness (35, 37, 44). Mode- and class-specific travel-time thresholds therefore followed the assumption that larger parks have wider potential service areas, whereas smaller parks mainly provide local services. Threshold selection also drew on research on realized service areas and human mobility (19, 45, 46). Walking thresholds for the four classes were 10, 15, 20, and 30 min, respectively. Cycling thresholds were 5, 10, 15, and 30 min, and driving thresholds were 5, 10, 15, and 30 min. A road-network dataset was constructed using GIS Pro 3.0. The multimodal thresholds followed network and decay treatments used in park-accessibility studies (36, 38).

Network travel times were calculated at 5 km/h for walking and 15 km/h for cycling. Driving speeds were assigned by road class: 75 km/h on motorways, 45 km/h on expressways, 30 km/h on arterial roads, and 20 km/h on local roads. The time thresholds delimited potential service areas but did not assign identical opportunities to all locations within them. A Gaussian distance-decay function was therefore applied so that accessibility weights declined smoothly with travel time and reached zero at the threshold (31).

### 2.3 Three-step floating catchment area method (3SFCA)

The three-step floating catchment area method (3SFCA) was used to estimate accessibility to park green spaces under supply-demand competition. The method extends the 2SFCA supply-demand framework by adding demand-point choice weights across candidate facilities (30, 34). Applications to green-space research have incorporated multimodal accessibility (36, 38), carrying-capacity constraints (47), and supply-demand equity assessment (48–50), thereby improving the method's planning relevance. Let *i* denote a population grid, *j* a park, and *m* a travel mode. The shortest network travel time is *t*_*ijm*_, and *T*_*jm*_ is the threshold for the corresponding park-size class and travel mode. The Gaussian distance-decay function within the threshold was defined as follows (Equation 1):


$$\begin{array}{l} f\left(t_{ijm}\right)\;=\;\frac{\exp\left[-1/2\left(\frac{t_{ijm}}{T_{jm}}\right)\right]\;-\;\exp\left(-1/2\right)}{1\;-\;\exp\left(-1/2\right)},\;0\;\leq \;t_{ijm}\;\leq \;T_{jm} \end{array}$$ (1)

Step 1. Park area *S*_*j*_ represented both supply volume and attractiveness. Choice weights were calculated within the set of candidate parks $J_{i}^{m}$ accessible to grid *i*:


$$\begin{array}{l} W_{ijm}\;=\;\frac{S_{j}f\left(t_{ijm}\right)}{\sum_{k\;\in \;J_{i}^{m}}S_{k}f\left(t_{ikm}\right)} \end{array}$$ (2)

Step 2. Population *Pop*_*i*_ was allocated to park *j* according to the choice weights. Competitive demand was calculated using Equation 3, and the park-area-to-demand ratio was calculated using Equation 4:


$$\begin{array}{l} D_{jm}\;=\;\underset{i\;\in \;I_{j}^{m}}{\sum }Pop_{i}W_{ijm} \end{array}$$ (3)

$$\begin{array}{l} R_{jm}\;=\;\frac{S_{j}}{D_{jm}} \end{array}$$ (4)

Step 3. Park area-to-demand ratios were allocated back to population units while retaining travel-time decay:


$$\begin{array}{l} A_{im}\;=\;\underset{j\;\in \;J_{i}^{m}}{\sum }W_{ijm}R_{jm}f\left(t_{ijm}\right) \end{array}$$ (5)

Here, *A*_*im*_ is 3SFCA accessibility for population grid *i* under travel mode *m*, expressed in m^2^/person. Choice weights for all parks were calculated from the same candidate set. Accessibility for the four park-size classes was then decomposed from total accessibility, allowing each class contribution to be interpreted directly as a component of the total. Park area therefore entered the model both as the supply quantity and as the scale component of the choice weight.

To test whether the linear area term imposed an excessively strong scale effect, we recalculated the model using four alternative transformations in the choice weight: $S_{j}^{0.5}$, log(1 + *S*_*j*_), ordinal weights of 1–4 for the four park-size classes, and min (*S*_*j*_, 250 ha). Actual park area was retained as the supply-capacity term in Equation 4 in all scenarios. We compared population-weighted accessibility, the population-weighted Gini coefficient, the spatial correlation of log-transformed accessibility, and the overlap of IPA Priority grids with the baseline model.

The Gaussian terms in Equations 2 and 5 represent two linked but distinct processes. In Equation 2, distance decay allocates each population grid among competing candidate parks. In Equation 5, it discounts the park supply-demand ratio when accessible opportunities are allocated back to residents. Because their combined use places greater emphasis on nearby service, we additionally estimated a single-decay specification in which the final *f*(*t*_*ijm*_) term was removed from Equation 5, while all other inputs and parameters were held constant.

### 2.4 Population-weighted zonal aggregation and distributional equity

3SFCA first generated accessibility *A*_*ims*_ for each population grid, where *s* denotes all parks or a particular park-size class. To compare the urban core, urban fringe, and suburb, a population-weighted zonal mean was calculated using Equation 6 instead of an unweighted arithmetic mean across grids:


$$\begin{array}{l} {\bar{A}}_{rms}\;=\;\frac{\sum_{i\;\in \;I_{r}}Pop_{i}A_{ims}}{\sum_{i\;\in \;I_{r}}Pop_{i}} \end{array}$$ (6)

Here, *I*_*r*_ is the set of population grids in zone *r*, and *Pop*_*i*_ is grid population. This indicator represents the mean area-based accessibility available to a randomly selected resident of the zone and remains expressed in m^2^/person. Population entered the analysis twice but served different roles. In Step 2 of 3SFCA, *Pop*_*i*_ determined the competitive demand faced by each park and directly affected grid accessibility. During zonal aggregation, *Pop*_*i*_ served only as a statistical weight, allowing more populous grids to represent more residents without altering any grid-level *A*_*ims*_.

Population was used at two linked stages for different analytical purposes. In the 3SFCA calculation, *Pop*_*i*_ represents competitive demand and contributes to the park-specific supply-demand ratio and the resulting accessibility *A*_*ims*_. In LISA and IPA, population is not treated as an independent validation variable; it represents the number or concentration of residents exposed to a modeled accessibility condition and the planning importance of that condition. The later analyses are therefore downstream diagnostics and are not statistically independent of the accessibility calculation. They identify modeled demand-accessibility mismatch and affected population rather than independently testing a causal relationship between population and accessibility.

Distributional equity was evaluated using population-weighted Lorenz curves and Gini coefficients (39). Grids were ranked by *A*_*ims*_ from low to high. *X*_*i*_ denotes the cumulative population share, and *Y*_*i*_ denotes the cumulative share of population × accessibility. A greater departure from the line of equality indicates a stronger concentration of accessible opportunities. The Gini coefficient was calculated using Equation 7:


$$\begin{array}{l} G\;=\;1\;-\;\overset{n}{\underset{i\;=\;1}{\sum }}\left(Y_{i}\;+\;Y_{i-1}\right)\left(X_{i}\;-\;X_{i-1}\right) \end{array}$$ (7)

### 2.5 Local bivariate spatial autocorrelation and IPA

Zonal means describe overall accessibility, whereas Gini coefficients describe its population distribution. Neither identifies where high demand is spatially associated with low accessibility. Grid population was therefore treated as the local variable and neighboring accessibility as the spatially lagged variable. Local bivariate Moran's I was calculated using GeoDa (51):


$$\begin{array}{l} I_{i}^{\left(P,A\right)}\;=\;z\left(Pop_{i}\right)\underset{j}{\sum }w_{ij}z\left(A_{jms}\right) \end{array}$$ (8)

Here, *w*_*ij*_ is a row-standardized *K*-nearest-neighbor spatial weight with *K* = 8. Directional pseudo-*p* values were obtained from 999 random permutations. A significant high-population–low-accessibility cluster denotes a high-population grid surrounded by low-accessibility neighbors.

IPA was derived from importance-performance analysis (42). For each travel mode and park-size class, the citywide median population of inhabited grids was used as the demand threshold. The citywide median accessibility among grids with positive accessibility was used as the performance threshold. The same thresholds were applied to the urban core, urban fringe, and suburb. High-demand–low-accessibility grids were classified as Priority, whereas high-demand–high-accessibility grids were classified as Maintain. Low-demand–high-accessibility grids were classified as Potential surplus, and low-demand–low-accessibility grids were classified as Low priority. Common thresholds ensured comparability among the three zones. IPA identified planning priorities, whereas local bivariate LISA identified statistically significant spatial clusters through permutation testing.

## 3 Results

### 3.1 Multimodal 3SFCA accessibility and differences among park-size classes

The alternative area transformations produced limited changes in citywide population-weighted accessibility, ranging from 0.24% to 4.93% relative to the linear-area baseline. The correlations of log-transformed grid accessibility with the baseline ranged from 0.974 to 1.000, and the Jaccard overlap of IPA Priority grids ranged from 0.881 to 0.999. Under every transformation and travel mode, population-weighted accessibility retained the same zonal ordering: suburb > urban fringe > urban core. These results indicate that the main spatial gradient and priority pattern were not driven solely by the linear area specification, although the functional form affected absolute values and some local classifications (Tables 1 and 2).

**Table 1:** Zonal population-weighted accessibility and ranking.

Scenario	Mode	Beijing	Core	Fringe	Suburb	Zonal ranking
linear area	Walking	9.724	5.445	12.030	18.234	Suburb>Fringe>Core
	Cycling	13.471	8.806	15.669	23.048	Suburb>Fringe>Core
	Driving	15.197	9.333	16.813	28.328	Suburb>Fringe>Core
square root	Walking	9.899	5.575	12.261	18.467	Suburb>Fringe>Core
	Cycling	13.898	9.293	15.976	23.437	Suburb>Fringe>Core
	Driving	15.572	9.750	17.265	28.524	Suburb>Fringe>Core
logarithm	Walking	10.019	5.662	12.377	18.675	Suburb>Fringe>Core
	Cycling	14.127	9.439	16.185	23.895	Suburb>Fringe>Core
	Driving	15.779	9.858	17.676	28.787	Suburb>Fringe>Core
class weights 1-4	Walking	9.982	5.632	12.362	18.596	Suburb>Fringe>Core
	Cycling	14.136	9.526	16.157	23.744	Suburb>Fringe>Core
	Driving	15.763	9.921	17.513	28.713	Suburb>Fringe>Core
250-ha cap	Walking	9.748	5.472	12.028	18.274	Suburb>Fringe>Core
	Cycling	13.867	9.441	15.752	23.147	Suburb>Fringe>Core
	Driving	15.554	9.899	17.055	28.269	Suburb>Fringe>Core

**Table 2:** Citywide equity and spatial stability.

Scenario	Mode	Δ Accessibility (%)	Gini	Priority (%)	Priority Jaccard	Log accessibility / r
linear area	Walking	0.00	0.857	60.62	1.000	1.000
	Cycling	0.00	0.626	56.35	1.000	1.000
	Driving	0.00	0.481	55.49	1.000	1.000
square root	Walking	1.80	0.859	60.64	0.981	0.999
	Cycling	3.17	0.635	56.79	0.953	0.997
	Driving	2.47	0.497	56.82	0.937	0.993
logarithm	Walking	3.03	0.862	60.68	0.961	0.994
	Cycling	4.87	0.647	57.36	0.888	0.984
	Driving	3.83	0.513	57.90	0.881	0.974
class weights 1-4	Walking	2.65	0.861	60.68	0.972	0.997
	Cycling	4.93	0.645	57.12	0.916	0.990
	Driving	3.72	0.510	57.26	0.907	0.982
250-ha cap	Walking	0.24	0.858	60.64	0.999	1.000
	Cycling	2.94	0.636	56.43	0.991	0.999
	Driving	2.35	0.498	56.08	0.966	0.993

Removing the final decay term increased citywide population-weighted accessibility by 134.55% for walking, 116.61% for cycling, and 93.99% for driving. Despite this marked change in absolute magnitude, the correlations of log-transformed accessibility between the single- and double-decay specifications remained 0.935–0.941. Priority-grid Jaccard overlap ranged from 0.831 to 0.873, while the population-weighted Gini coefficient changed from 0.857 to 0.846 for walking, from 0.626 to 0.637 for cycling, and from 0.481 to 0.483 for driving. The double-decay formulation therefore strongly affected the absolute scale of accessibility but preserved the broad spatial pattern and much, although not all, of the priority classification (Table 3).

**Table 3:** Single- double-decay comparison.

Scenario	Mode	Accessibility (m^2^/person)	^Δ^ Accessibility (%)	Gini	Priority Jaccard	Log accessibility / r
Double decay	Walking	9.724	0.00	0.857	1.000	1.000
Double decay	Cycling	13.471	0.00	0.626	1.000	1.000
Double decay	Driving	15.197	0.00	0.481	1.000	1.000
Single decay	Walking	22.807	134.55	0.846	0.867	0.935
Single decay	Cycling	29.180	116.61	0.637	0.831	0.941
Single decay	Driving	29.481	93.99	0.483	0.873	0.941

Table 4 shows opposing gradients in park supply and population demand across the three zones. The urban core contained 56.2% of Beijing's population but only 13,822.7 ha of park green space. The urban fringe and suburb contained 21.4% and 22.5% of the population, respectively, but 23,420.6 and 23,049.4 ha of park green space. Park-size structure also differed substantially by zone. District green spaces dominated the urban core, accounting for 63.6% of its park area. Regional green spaces dominated the urban fringe, also accounting for 63.6%. District and regional green spaces together accounted for 89.1% of park area in the suburb.

**Table 4:** Population demand and park green-space supply structure across the three urban zones.

Urban zone	Population (10,000 persons)	Population share	Population grids	Parks	Park area (ha)	Area composition (small and micro/community/district/regional)
Urban core	1079.1	56.2%	3997	570	13,822.7	8.1%/15.9%/63.6%/12.4%
Urban fringe	410.7	21.4%	2326	225	23,420.6	1.3%/3.8%/31.3%/63.6%
Suburb	431.3	22.5%	3765	378	23,049.4	2.7%/8.2%/45.0%/44.1%

Given these supply-demand structures, the citywide population-weighted mean accessibility values for walking, cycling, and driving were 9.72, 13.47, and 15.20 m^2^/person, respectively. All three modes showed a consistent outward-increasing gradient. Walking, cycling, and driving accessibility was 5.44, 8.81, and 9.33 m^2^/person in the urban core. The corresponding values were 12.03, 15.67, and 16.81 m^2^/person in the urban fringe and 18.23, 23.05, and 28.33 m^2^/person in the suburb. Cycling and driving increased mean accessibility in every zone but did not change the zonal ranking, with the urban core remaining lowest and the suburb highest. Spatial high-value areas were concentrated around park-rich locations on the urban periphery (Figure 4).

**Figure 4:**
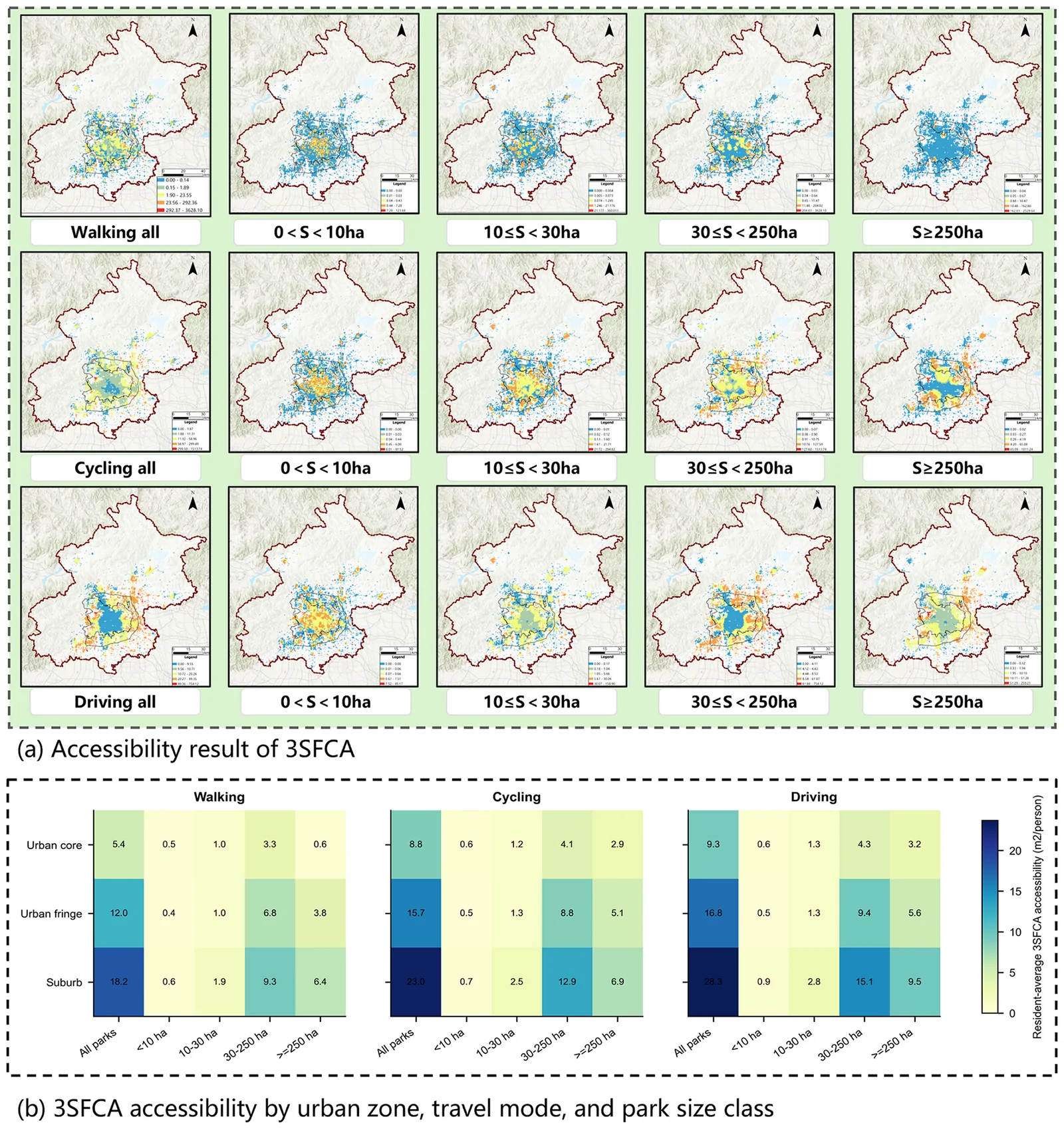
Spatial distribution and summary statistics of 3SFCA accessibility by urban zone, travel mode, and park-size class. **(a)** Spatial distribution of 3SFCA accessibility. **(b)** Summary statistics. “Basemap: Esri World Topographic Map (ArcGIS Online), used under Esri's Terms of Use for Static Maps. Copyright © 2026 Esri and its licensors. All rights reserved.” The service-layer credits shown for the maps include Esri, © OpenStreetMap contributors, TomTom, Garmin, FAO, METI/NASA, USGS, NOAA, and CGIAR.

When accessibility was decomposed by park-size class, district green spaces made the largest contribution in every zone and travel mode. Their contributions ranged from 3.33 to 4.29 m^2^/person in the urban core and from 6.80 to 9.44 m^2^/person in the urban fringe. The corresponding range in the suburb was 9.32–15.12 m^2^/person. Contributions from regional green spaces increased as travel ranges expanded. From walking to driving, they rose from 0.59 to 3.16 m^2^/person in the urban core. The corresponding urban-fringe increase was from 3.83 to 5.58 m^2^/person, and the suburban increase was from 6.42 to 9.51 m^2^/person. Across all combinations, small and micro green spaces and community green spaces contributed 0.39–0.90 and 1.00–2.80 m^2^/person, respectively. District green spaces consistently accounted for the largest contribution share, whereas the share of regional green spaces was generally higher under cycling and driving than under walking (Figure 5).

**Figure 5:**
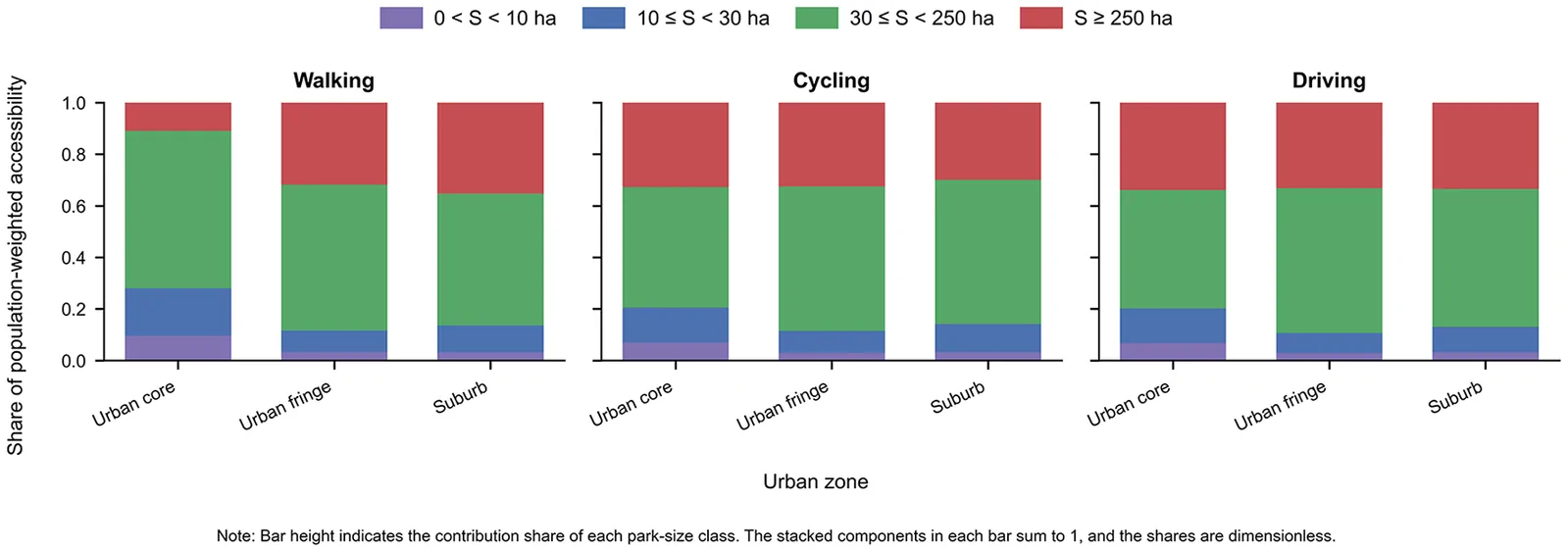
Contributions of different park-size classes to 3SFCA accessibility by urban zone and travel mode.

Population-grid inclusion affected the magnitude of accessibility but left most of the focal spatial structure stable (Appendix A, Table A1 and Figure A1). When all positive-population grids were added only to the competitive-demand denominator, citywide population-weighted accessibility within the original focal evaluation set decreased by 19.34% for walking, 16.08% for cycling, and 16.00% for driving, confirming that excluding low-density residents reduced competitive demand and increased the original accessibility estimates. Nevertheless, Spearman correlations of log-transformed grid accessibility were 0.9997, 0.9956, and 0.9829, respectively. Jaccard overlap ranged from 0.931 to 0.994 for IPA Priority grids and from 0.921 to 0.981 for high-population-low-accessibility LISA grids. Under the strict all-positive specification, the urban core remained the lowest-accessibility zone for every mode, and cycling and driving retained the original zonal order. Walking, however, changed from suburb > urban fringe > urban core to urban fringe > suburb > urban core. Thus, the persistent disadvantage of the urban core was robust, whereas the walking comparison between the urban fringe and suburb was sensitive to the population-grid inclusion rule.

### 3.2 Zonal differences in Lorenz curves and Gini coefficients

Lorenz curves and Gini coefficients revealed distributional differences beyond mean accessibility (Figure 6). In all three zones, the walking curve departed furthest from the line of equality, whereas the driving curve was closest to it. The Gini coefficients for walking, cycling, and driving were 0.781, 0.619, and 0.424 in the urban core. They were 0.832, 0.434, and 0.279 in the urban fringe and 0.906, 0.637, and 0.462 in the suburb. Walking showed the greatest inequality in every zone, whereas driving showed the least. Notably, the suburb combined the highest mean accessibility with the highest walking Gini coefficient, indicating that a high accessibility level does not necessarily imply a more equitable population distribution.

**Figure 6:**
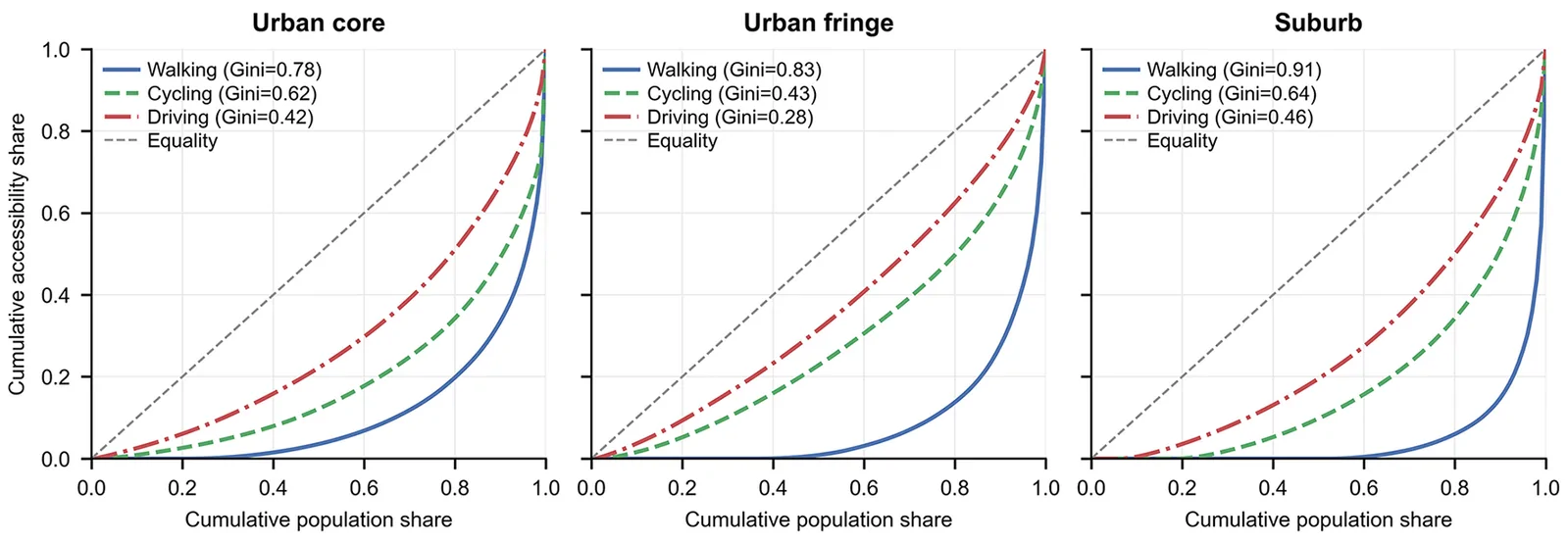
Lorenz curves and Gini coefficients for the three travel modes within each urban zone.

### 3.3 Local bivariate spatial autocorrelation

Global bivariate Moran's I was negative for all three travel modes (Table 5). The statistics were −0.084 for walking, −0.160 for cycling, and −0.238 for driving, with permutation *p*-values of 0.001 in all cases. High-population grids were therefore generally adjacent to low-accessibility grids, and this negative spatial association strengthened from walking to driving.

**Table 5:** Global bivariate Moran's I by travel mode.

Travel mode	Global bivariate Moran's I	Permutation *p*-value	Neighbors (K)	Permutations
Walking	−0.084	0.001	8	999
Cycling	−0.160	0.001	8	999
Driving	−0.238	0.001	8	999

Local bivariate LISA further identified significant high-population–low-accessibility clusters (Figure 7 and Table 6). The population shares in these clusters under walking, cycling, and driving were 24.4, 60.5, and 65.7% in the urban core. These values exceeded those in the urban fringe (15.6, 9.4, and 10.4%) and the suburb (4.1, 4.3, and 1.6%). Significant cycling and driving clusters formed largely continuous patterns in the urban core, whereas most grids in the urban fringe and suburb did not pass the local significance test.

**Figure 7:**
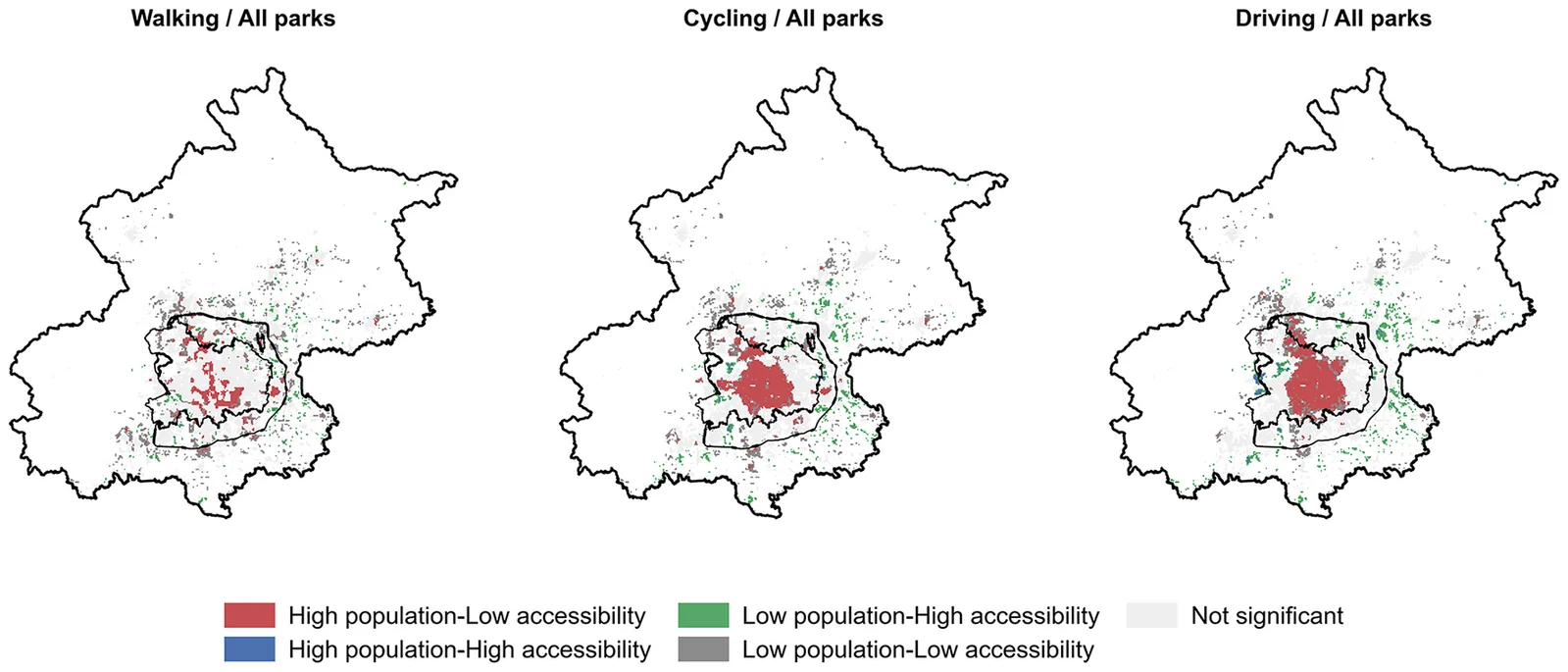
Local bivariate spatial autocorrelation between population demand and accessibility for all park green spaces.

**Table 6:** Local LISA results by urban zone and travel mode for all park green spaces.

Urban zone	Travel mode	High-population–low-accessibility grids	Population in high-population–low-accessibility clusters	Dominant LISA category	Population in dominant category
Urban core	Walking	16.1%	24.4%	Not significant	71.2%
Urban core	Cycling	39.4%	60.5%	High population–low accessibility	60.5%
Urban core	Driving	43.5%	65.7%	High population–low accessibility	65.7%
Urban fringe	Walking	8.7%	15.6%	Not significant	71.6%
Urban fringe	Cycling	4.9%	9.4%	Not significant	80.9%
Urban fringe	Driving	6.4%	10.4%	Not significant	79.6%
Suburb	Walking	2.0%	4.1%	Not significant	63.4%
Suburb	Cycling	2.0%	4.3%	Not significant	63.1%
Suburb	Driving	0.8%	1.6%	Not significant	62.8%

Decomposition by park-size class revealed different class-specific structures of significant high-population–low-accessibility clustering (Figure 8). In the urban core, clustered population shares for district and regional green spaces were 51.6% and 54.7% under cycling. These shares increased to 57.9% and 63.6% under driving. In the urban fringe, the highest shares under cycling and driving occurred for community green spaces, at 28.3% and 26.8%, respectively. Overall shares were lower in the suburb, although regional green spaces were still associated with 21.2% of the population in significant clusters under cycling.

**Figure 8:**
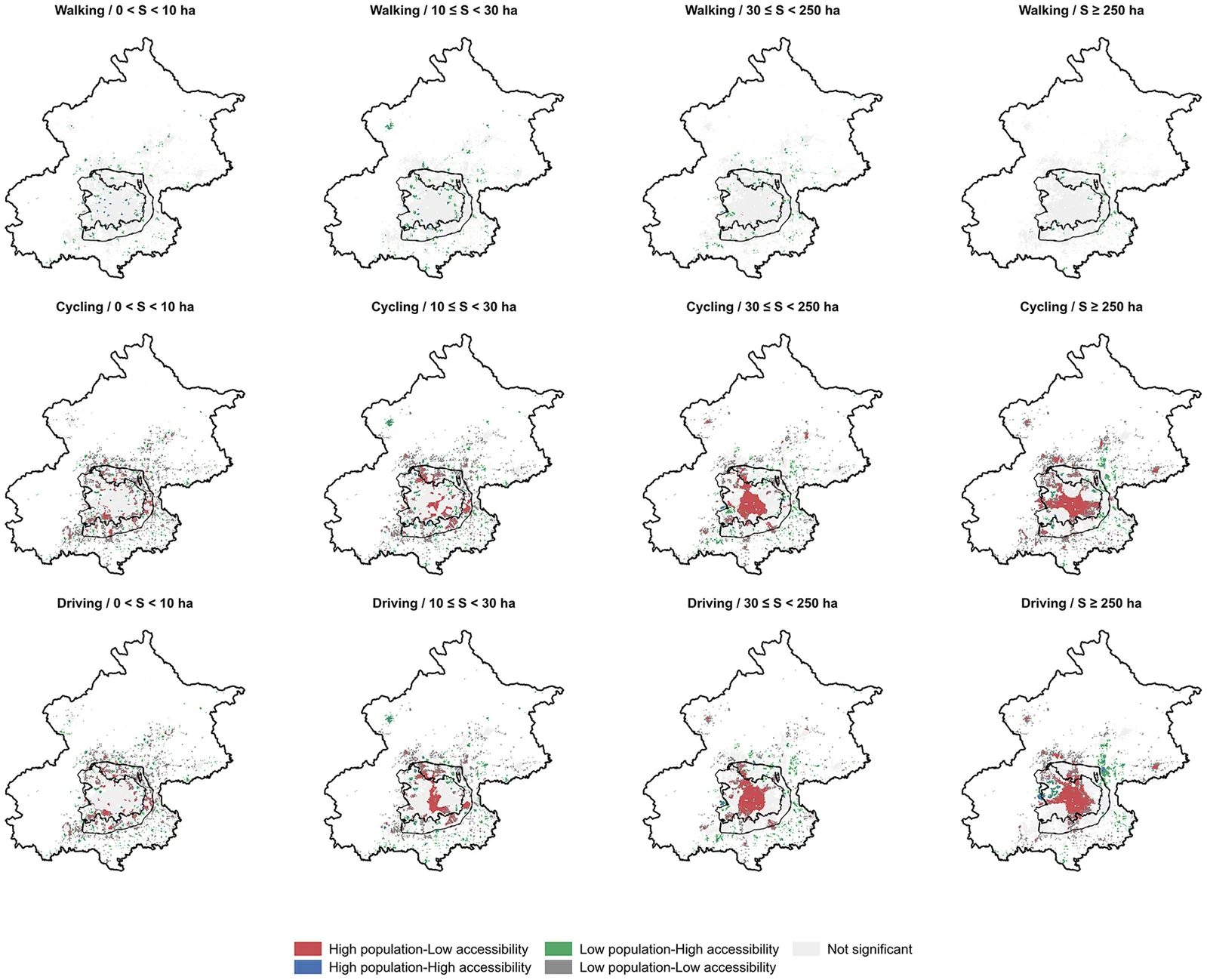
Local bivariate spatial autocorrelation between population demand and accessibility by park-size class.

### 3.4 IPA categories and their spatial distribution

Using common population-demand and accessibility thresholds, IPA classified grids as Priority, Maintain, Potential surplus, or Low priority. High-population grids below the accessibility threshold occupied the lower-right quadrant. Cycling and driving increased accessibility in some grids, but substantial numbers of Priority grids remained under all three modes (Figure 9).

**Figure 9:**
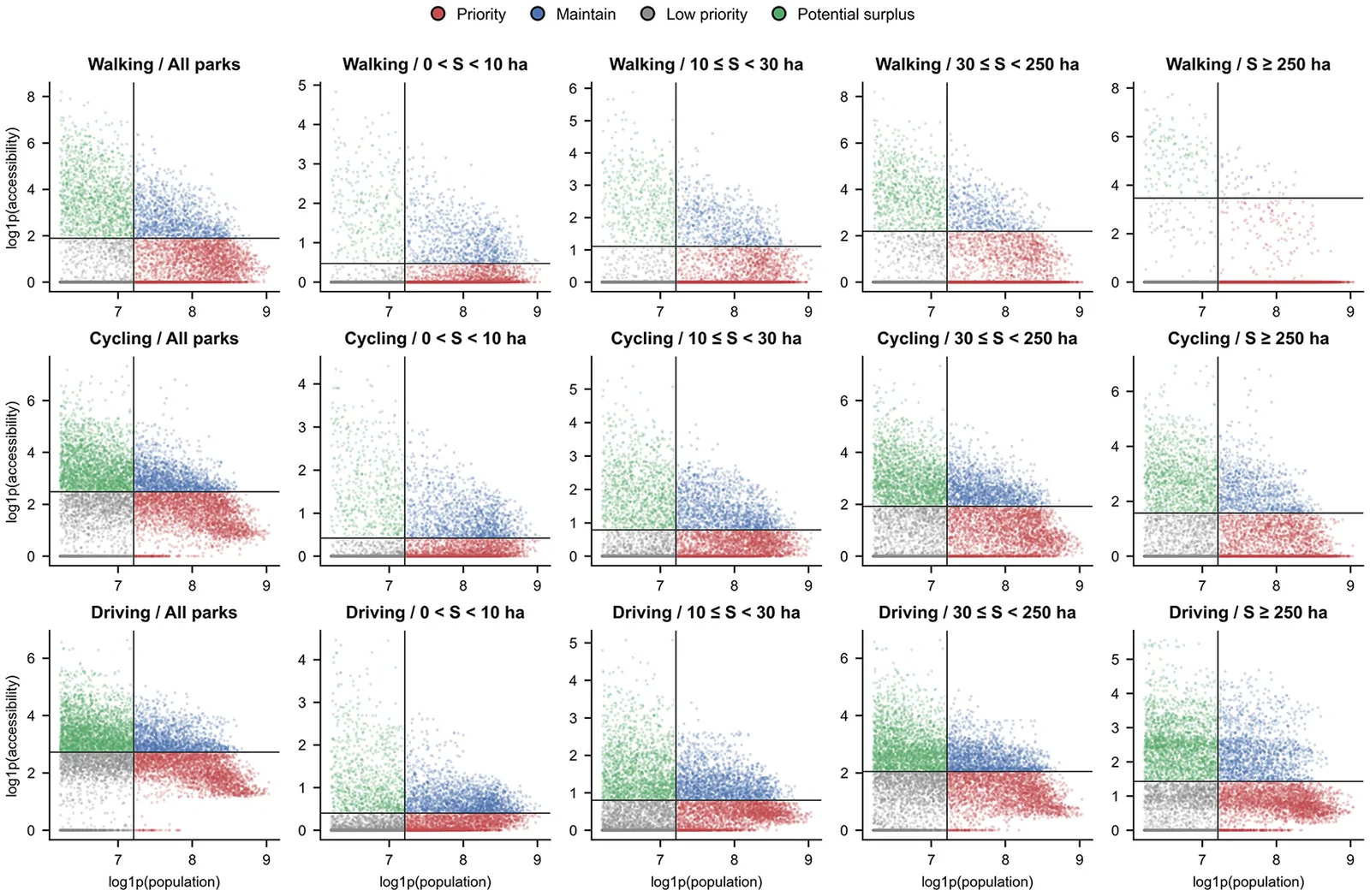
IPA quadrants based on population demand and accessibility.

In the urban core, Priority population shares under walking, cycling, and driving were 75.1, 77.5, and 78.4%, respectively (Table 7). Priority dominated all three modes. The corresponding shares in the urban fringe were 52.4, 37.1, and 38.8%; Maintain slightly dominated under cycling. In the suburb, the shares fell to 32.3, 21.8, and 14.0%. Low priority dominated walking in the suburb, whereas Maintain dominated driving, for which the Potential surplus population share reached 33.3%.

**Table 7:** Composition of IPA categories by urban zone and travel mode.

Urban zone	Travel mode	Dominant IPA category	Low priority (%)	Maintain (%)	Priority (%)	Potential surplus (%)
Urban core	Walking	Priority	5.7%	15.7%	75.1%	3.5%
Urban core	Cycling	Priority	4.4%	13.3%	77.5%	4.8%
Urban core	Driving	Priority	5.1%	12.4%	78.4%	4.1%
Urban fringe	Walking	Priority	15.9%	23.8%	52.4%	7.9%
Urban fringe	Cycling	Maintain	9.5%	39.1%	37.1%	14.3%
Urban fringe	Driving	Priority	8.5%	37.4%	38.8%	15.2%
Suburb	Walking	Low priority	40.1%	15.7%	32.3%	11.9%
Suburb	Cycling	Potential surplus	25.1%	26.2%	21.8%	26.9%
Suburb	Driving	Maintain	18.8%	34.0%	14.0%	33.3%

The spatial distribution of IPA categories was consistent with the zonal statistics (Figure 10). Priority grids were concentrated in the urban core and in high-population areas adjoining the urban fringe. Maintain, Potential surplus, and Low priority grids became more common toward the suburb. Driving expanded the distribution of high-accessibility grids, but the urban core retained a continuous Priority area.

**Figure 10:**
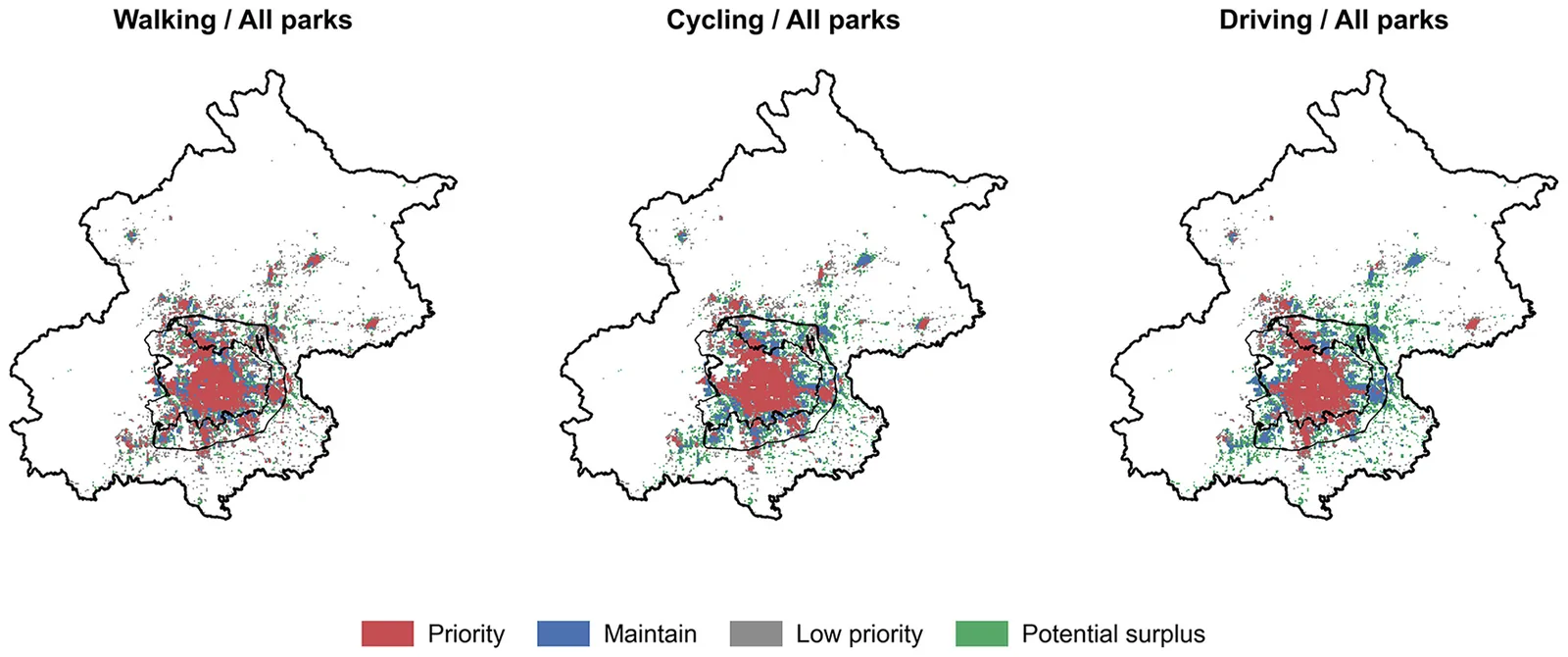
Spatial distribution of IPA categories for all park green spaces.

After decomposition by park-size class, the Priority category retained a clear zonal gradient (Figure 11). Under walking, Priority population shares across the four classes ranged from 69.7% to 90.6% in the urban core. The corresponding urban-fringe range was 64.0–74.4%. Under driving, however, the urban-fringe shares for district and regional green spaces declined to 32.5% and 30.6%, respectively. In the suburb, the corresponding driving shares across the four classes were only 14.0–25.2%. Differences among park-size classes were most pronounced under walking in the urban core and urban fringe and weakened substantially under driving in the suburb.

**Figure 11:**
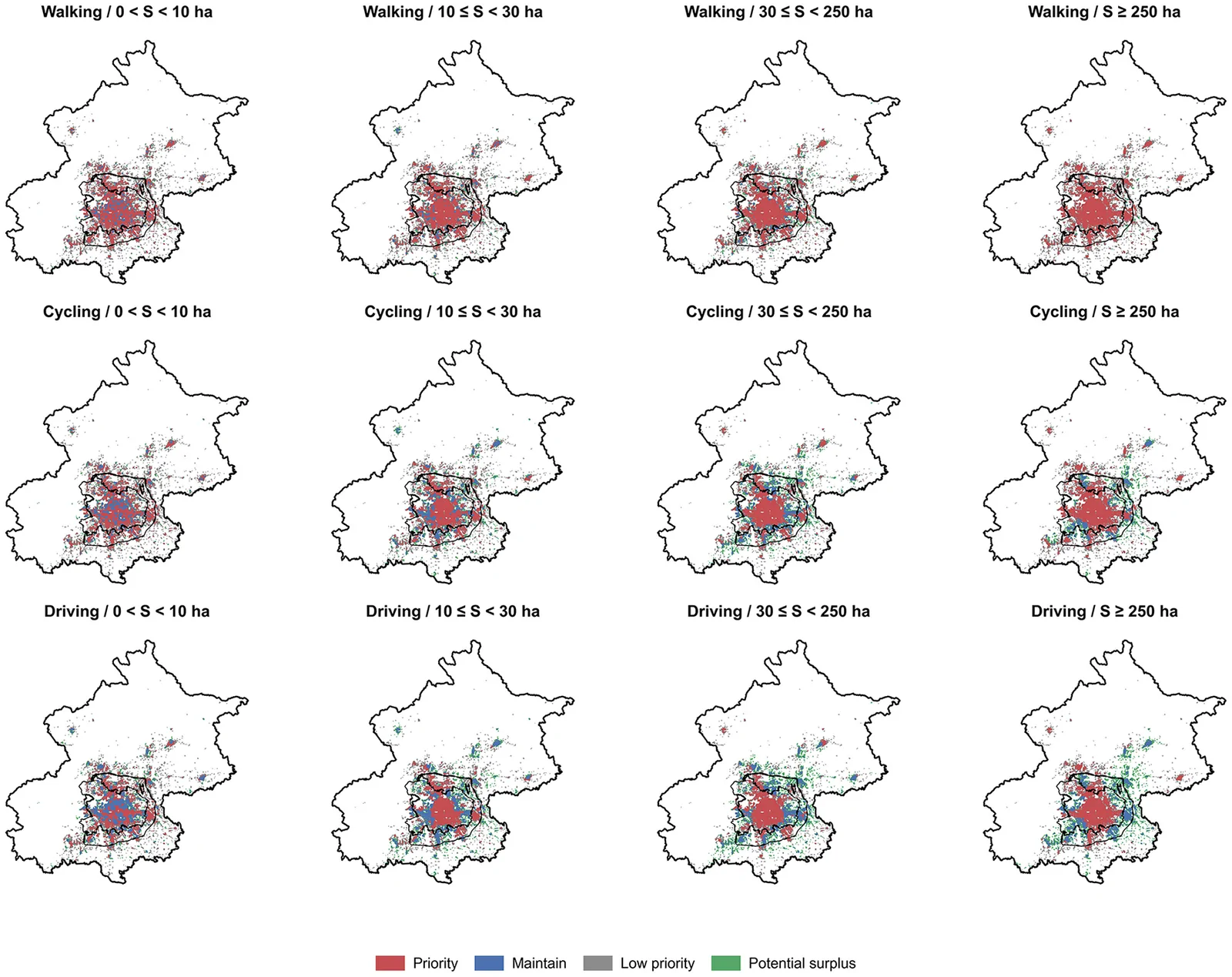
Spatial distribution of IPA categories by park-size class.

## 4 Discussion

Taken together, the results reveal clear spatial differentiation in equity in access to Beijing's park green spaces. Population-weighted mean accessibility increased from the urban core to the suburb, yet higher mean values did not imply a more equitable distribution. Park-size class and travel mode further shaped how green-space supply was converted into opportunities for residents. The observed core-periphery disparity was therefore not a simple difference in total green-space area. Instead, it reflected the joint effects of population demand intensity, park-size structure, and mobility.

The population-grid sensitivity analysis qualifies the outward-increasing accessibility gradient. The most stable regional finding is that the urban core consistently has the lowest accessibility. The relative walking advantage of the suburb over the urban fringe should not be generalized across population-grid specifications because it reversed when all positive-population grids were included as both demand and evaluation units. Cycling and driving showed more stable zonal rankings.

### 4.1 Accessibility differences among the urban core, urban fringe, and suburb across travel modes

The principal constraint in the urban core was the persistent dilution of limited park supply by high population demand. This zone contained 56.2% of Beijing's population but only 13,822.7 ha of park green space. Even when driving expanded the search range, population-weighted mean accessibility increased only from 5.44 m^2^/person under walking to 9.33 m^2^/person. Moreover, 75.1–78.4% of the population remained in the IPA Priority category. This pattern is consistent with the supply pressure identified by studies of park distribution and environmental justice in central Beijing (13, 14). Faster travel was therefore insufficient to offset competition generated by concentrated demand, producing a characteristic pattern of demand-induced supply dilution in the urban core.

The urban fringe showed a transitional pattern in which growth in aggregate supply was not matched by local service provision. Its park area reached 23,420.6 ha, of which regional green spaces accounted for 63.6%. Cycling and driving accessibility was consequently much higher than in the urban core. Nevertheless, 52.4% of the population remained in the walking Priority category, indicating that increased park area had not been converted into walkable community services. This finding accords with previous evidence of inadequate public-service accessibility in Beijing's urban fringe and asynchronous opportunity distribution during urban expansion (12, 16). The principal constraint may lie in connections among new residential clusters, park entrances, and active-travel networks.

The suburb combined high area-based accessibility with a dispersed distribution of walking opportunities. District and regional green spaces accounted for 89.1% of park area, and mean driving accessibility reached 28.33 m^2^/person. At the same time, the walking Gini coefficient was as high as 0.906. Research on park dynamics and realized service areas in Beijing also emphasizes population location and actual activity ranges (17, 19). These factors determine whether large peripheral supplies are converted into resident opportunities. The aggregate advantage in the suburb was therefore realized mainly at larger spatial scales and under faster travel. It did not guarantee equitable daily walking opportunities for residents of suburban towns.

The three zones consequently did not represent a single form of green-space shortage that simply declined from the center outward. The urban core showed high-demand dilution, whereas the urban fringe showed incomplete conversion of supply growth into local services. The suburb combined an aggregate advantage with dispersed walking opportunities. Population-weighted zonal means distinguished spatially high accessibility values from the average opportunities available to residents. Gini coefficients, local bivariate LISA, and IPA then revealed distributional and clustering problems masked by zonal averages. Higher mean accessibility in the urban fringe and suburb should therefore not be interpreted directly as equitable daily walking service.

### 4.2 Multimodal accessibility differences among park-size classes

Park-size classes and travel modes jointly formed a complementary service structure. District green spaces (30–250 ha) made the largest contribution in all three zones and travel modes, forming the principal backbone of area-based accessibility. The contribution of regional green spaces (≥250 ha) increased markedly from walking to cycling and driving. Their cross-zone service therefore depends more strongly on wider activity ranges and road connections. Small and micro green spaces and community green spaces made smaller area-based contributions because park area represented supply and because these classes had shorter service thresholds. Their lower contributions should not be interpreted as lower actual use. Instead, they reflect the limited representation of local service potential within an area-based metric.

This pattern is consistent with multimodal 3SFCA research in Shanghai and park-classification research in Wuhan (37, 38). Other supply-demand studies also show that park size and travel mode jointly shape accessibility (50). Multimodal park research indicates that a wider range of travel options can alter the catchments of parks at different service levels (35, 36). The present findings further indicate that the same park-size class has different planning implications across urban zones. In the urban core, small and micro green spaces and community green spaces determine whether short-distance opportunities are embedded in high-demand neighborhoods. The urban fringe requires district green spaces as a service backbone, connected to new residential clusters through park entrances and active-travel networks. Regional green spaces in the suburb support cross-zone recreation but cannot replace smaller-scale provision within suburban towns. Park-size class is therefore not merely a quantitative classification; it also defines a service hierarchy linking regional demand to travel mode.

### 4.3 Optimization priorities and strategies by urban zone

IPA and local bivariate LISA provided complementary evidence of a centrally concentrated pattern from the perspectives of planning priority and spatial significance. IPA used common citywide median thresholds to identify high-demand–low-accessibility grids for planning without testing spatial significance. Local bivariate LISA instead tested whether high-population grids were significantly adjacent to low-accessibility grids. In the urban core, IPA Priority population shares were 77.5% under cycling and 78.4% under driving. The corresponding population shares in significant high-population–low-accessibility clusters reached 60.5% and 65.7%. Both forms of evidence consistently identified the urban core as the principal intervention area. The urban fringe had lower significant LISA shares, but its walking Priority population share remained 52.4%, indicating widespread local-service deficits that did not necessarily form significant clusters. Significant clustering was generally limited in the suburb, where localized shortfalls in suburban towns were obscured by high zonal means.

Planning strategies should consequently distinguish among urban zones, park-size classes, and travel modes. The urban core requires additional small and micro green spaces and community green spaces, shared use of school, institutional, and existing green-space edges, and greater entrance density and active-travel permeability. In the urban fringe, district green spaces should form the service backbone, supported by connections among residential clusters, rail stations, greenways, and regional-park entrances. The suburb should protect the ecological and recreational functions of regional green spaces while concentrating new local provision in suburban towns and densely populated villages. This sequence responds to the different concerns revealed by zonal means, population-level distributional equity, and significant spatial clustering. It also helps translate spatial diagnosis into differentiated planning actions for health-supportive environments.

### 4.4 Limitations

Multimodal rail-transit research in Beijing has shown that faster travel modes can improve overall accessibility and its distribution (52). We similarly observed that driving increased mean accessibility and reduced Gini coefficients in all three zones. However, the urban core retained high shares of both the IPA Priority population and significant high-population–low-accessibility clusters under driving. These findings are not contradictory because a wider service range does not remove the competitive allocation of park area under concentrated population demand. Research on public-transport access to large parks has likewise shown that extending transport reach does not automatically eliminate group disparities (53). Improving transport connectivity and increasing local green-space provision should therefore be treated as complementary rather than substitutable strategies.

This study has three limitations. First, the sensitivity results support the spatial robustness of the diagnosis but do not validate park area as an empirical measure of recreational attractiveness. Visitor counts, park-quality indicators, amenity POIs, and observed mobility should be used in future work to calibrate the choice component. Population-grid inclusion affected both competitive demand and the composition of the evaluation sample. Adding all positive-population grids to competitive demand reduced population-weighted accessibility within the focal evaluation set by 16.00–19.34%, showing that the baseline absolute values were sensitive to the exclusion of low-density demand. When all populated grids were also included as evaluation units, the urban core remained lowest for every mode, but walking accessibility was higher in the urban fringe (19.91m^2^/person) than in the suburb (17.33 m^2^/person). Accordingly, the urban-core disadvantage is robust, whereas absolute accessibility and the walking comparison between the urban fringe and suburb should be interpreted as sensitive to population-grid inclusion.

Second, the results are sensitive to the spatial scales and representations of park boundaries, population grids, road networks, and travel-time thresholds (54). Because the park access points were not derived from verified public-entrance data, this approximation may underestimate actual travel time for some parks, particularly large parks, forest parks, and wetland parks with restricted boundaries or few public entrances. Future research should incorporate official entrance locations, opening hours, and field-survey data to refine the network-accessibility estimates. Third, public transport was not included, and driving speeds assigned by road class remain a planning-scale approximation. Future research should test sensitivity to grid size and travel-time thresholds. Park entrances, opening hours, resident surveys, and human-mobility data should be combined to validate the realized service ranges of different park-size classes. The supplementary role of non-park green space in accessibility equity should also be examined (51, 55).

## 5 Conclusions

This study developed a multimodal framework for comparing equity in accessibility to park green spaces across the urban core, urban fringe, and suburb. Grid-level 3SFCA measured accessibility under supply-demand competition. Population-weighted zonal means, Lorenz curves, and Gini coefficients characterized overall levels and distributional differences, while local bivariate LISA and IPA identified significant spatial clusters and planning priorities.

Equity in access to Beijing's park green spaces was jointly shaped by regional population demand, park-size class, and travel mode. Across the tested population-grid specifications, the urban core consistently remained the lowest-accessibility zone, whereas the relative walking order of the urban fringe and suburb was sensitive to whether all positive-population grids were included in the evaluation set. The urban core exhibited demand-induced supply dilution, whereas the urban fringe combined supply growth with inadequate local service. The suburb combined high mean area-based accessibility with an unequal distribution of walking opportunities. District green spaces formed the most stable area-based service backbone across all three zones. Regional green spaces mainly supported cross-zone access through cycling and driving. Small and micro green spaces and community green spaces served short-distance needs in the urban core and urban fringe. Both the IPA Priority population and significant high-population–low-accessibility LISA clusters were concentrated in the urban core. The urban fringe was characterized by deficits in local walking service, whereas localized shortfalls in suburban towns were obscured by high zonal means.

Park planning in Beijing should therefore avoid a uniform area-compensation approach. The urban core requires more local green spaces together with improved entrances and active-travel connections. The urban fringe requires stronger links between district green spaces and expanding residential areas. The suburb requires additional local provision in towns while regional green spaces are protected. Only by integrating urban spatial gradients, park-size classes, and travel modes within a common supply-demand framework can aggregate green-space resources be converted more effectively into opportunities available to residents.

## Data Availability

The data analyzed in this study is subject to the following licenses/restrictions. The data supporting the findings of this study are available from the corresponding author upon reasonable request. Requests to access these datasets should be directed to Haiyuan Quan, quanhaiyuan@tju.edu.cn.

## References

[1] Maas J Verheij RA Groenewegen PP de Vries S Spreeuwenberg P. Green space, urbanity, and health: how strong is the relation? J Epidemiol Community Health. (2006) 60:587–92. doi: 10.1136/jech.2005.043125 PMC2566234 16790830

[2] Wolch RJ. Byrne, Jason, Newell PJ. Urban green space, public health, and environmental justice: the challenge of making cities ‘just green enough' Landscape and Urban Planning. (2014) 125:234–44. doi: 10.1016/j.landurbplan.2014.01.017

[3] Ekkel Dinand E Vries de Sjerp. Nearby green space and human health: evaluating accessibility metrics. Landsc Urban Plan. (2017) 157:214–20. doi: 10.1016/j.landurbplan.2016.06.008

[4] Higgs G Fry R Langford M. Investigating the implications of using alternative GIS-based techniques to measure accessibility to green space. Environ Plann B: Plan Des. (2012) 39:326–43. doi: 10.1068/b37130

[5] Chen B Wu S Song Y Webster C Xu B Gong P. Contrasting inequality in human exposure to greenspace between cities of Global North and Global South. Nat Commun. (2022) 13:4636. doi: 10.1038/s41467-022-32258-4 35941122 PMC9360024

[6] Dai D. Racial/ethnic and socioeconomic disparities in urban green space accessibility: Where to intervene? Landsc Urban Plan. (2011) 102:234–44. doi: 10.1016/j.landurbplan.2011.05.002

[7] Xing L Liu Y Wang B Yiheng W Haijiang L. An environmental justice study on spatial access to parks for youth by using an improved 2SFCA method in Wuhan, China. Cities (2020) 96:102405. doi: 10.1016/j.cities.2019.102405

[8] Rigolon A. A complex landscape of inequity in access to urban parks: A literature review. Landsc Urban Plan. (2016) 153:160–69. doi: 10.1016/j.landurbplan.2016.05.017

[9] Jiang Y Sun Z Lu Y. Revealing the spatiotemporal pattern of urban vibrancy at the urban agglomeration scale: Evidence from the Pearl River Delta, China. Applied Geography (2025) 181:103694. doi: 10.1016/j.apgeog.2025.103694

[10] Jiang Y Chen L Lu Y. Spatial disparity of individual and collective walking behaviors: a new theoretical framework. Transportation Research Part D: Transport and Environment (2021) 101:103096. doi: 10.1016/j.trd.2021.103096

[11] Chen Y Gu Y Cao L Jiang Y. Urban heat resilience from a state–process perspective: heatwave-induced cooling service supply–demand mismatch and hierarchical spatial allocation of greening. Sustain Cities Soc. (2026) 148:107593. doi: 10.1016/j.scs.2026.107593

[12] Zhao P W J. Land use and travel burden of residents in urban fringe and rural areas: an evaluation of urban-rural integration initiatives in Beijing. Land Use Policy (2021) 103:105309. doi: 10.1016/j.landusepol.2021.105309

[13] Feng S Chen L Sun R . The distribution and accessibility of Urban Parks in Beijing, China: implications of social equity. Int J Environ Res Public Health. (2019) 16:4894. doi: 10.3390/ijerph16244894 PMC6950166 31817222

[14] Cao Y Guo Y Zhang M. Research on the equity of urban green park space layout based on Ga2SFCA optimization method—taking the core area of Beijing as an example. Land. (2022) 11:1323. doi: 10.3390/land11081323

[15] Qin J Tong S Wang Q. Exploring the disparities in urban park accessibility from multiple demand perspectives: a case study in Beijing, China. Ecolog Indic. (2024) 163:112124. doi: 10.1016/j.ecolind.2024.112124

[16] Huang Y Lin T Zhang G Jones L Xue X Ye H . Spatiotemporal patterns and inequity of urban green space accessibility and its relationship with urban spatial expansion in China during rapid urbanization period. Sci Total Environ. (2022) 809:151123. doi: 10.1016/j.scitotenv.2021.151123 34699811

[17] Kong X Sun Y Xu C. Effects of urbanization on the dynamics and equity of access to urban parks from 2000 to 2015 in Beijing, China. Forests. (2021) 12:1796. doi: 10.3390/f12121796

[18] Tian D Wang J Xia C Jian Z Jun Z Zihao T . The relationship between green space accessibility by multiple travel modes and housing prices: a case study of Beijing. Cities. (2024) 145:104694. doi: 10.1016/j.cities.2023.104694

[19] Guo S Yang G Pei T Ting M Ci S Hua S . Analysis of factors affecting urban park service area in Beijing: Perspectives from multi-source geographic data. Landsc Urban Plan. (2019) 181:103–17. doi: 10.1016/j.landurbplan.2018.09.016

[20] Guo S Song C Pei T Yaxi L Ting M Yunyan D . Accessibility to urban parks for elderly residents: perspectives from mobile phone data. Landsc Urban Plan. (2019) 191:103642. doi: 10.1016/j.landurbplan.2019.103642

[21] Zhu W Liao T Zeng B Zhu L Chen P. Rethinking urban park equity: a people-centered assessment of supply–demand mismatch using mobile phone data. Sustainability. (2026) 18:4541. doi: 10.3390/su18094541

[22] Chen J Liu B Feng Y. Actual supply-demand of the urban green space in a populous and highly developed city: evidence based on mobile signal data in Guangzhou. Ecol Indic. (2024) 169:112839. doi: 10.1016/j.ecolind.2024.112839

[23] Yan L Jin X Zhang J. Equity in park green spaces: a bibliometric analysis and systematic literature review from 2014–2023. Front Environ Sci. (2024) 12:1374973. doi: 10.3389/fenvs.2024.1374973

[24] Cai Z Lan H Ouyang Z . A comprehensive assessment of urban greenspace accessibility and supply-demand characteristics in Beijing. Environ Sustain Indic. (2026) 31:101394. doi: 10.1016/j.indic.2026.101394

[25] Ding T Wang C Zeng B Yuqi L Yunyuan L. Accessibility and social equity of Urban park green spaces in megacities from an environmental justice perspective: a case study of the six central Districts of Beijing. Land. (2026) 15:484. doi: 10.3390/land15030484

[26] Yuzhen Z Jie W Yang C Jianping Y. An assessment of urban parks distribution from multiple dimensions at the community level: a case study of Beijing. Environ Impact Assess Rev. (2021) 91:106663. doi: 10.1016/j.eiar.2021.106663

[27] Zhang Y Zhang J Shi X . Accessibility analysis and optimization strategies of green spaces for enhancing climate resilience: equity-oriented insights from megacities. Environ Sci Policy. (2025) 173:104234. doi: 10.1016/j.envsci.2025.104234

[28] Zheng X Huang Y. Diagnosing urban green space inequality: multi-source data and explainable modeling of park perceptions and service accessibility in Beijing. Humanit. Soc Sci Commun. (2026) 13:1466. doi: 10.1057/s41599-026-07794-9

[29] Zhang X Lu H Holt BJ. Modeling spatial accessibility to parks: a national study. Int J Health Geogr. (2011) 10:31. doi: 10.1186/1476-072X-10-31 PMC3116453 21554690

[30] Luo W Wang F. Measures of spatial accessibility to health care in a GIS environment: synthesis and a case study in the Chicago region. Environ Plan B, Plann Des. (2003) 30:865–84. doi: 10.1068/b29120 PMC8238135 34188345

[31] Luo W Qi Y. An enhanced two-step floating catchment area (E2SFCA) method for measuring spatial accessibility to primary care physicians. Health Place. (2009) 15:1100–07. doi: 10.1016/j.healthplace.2009.06.002 19576837

[32] McGrail RM Humphreys SJ. Measuring spatial accessibility to primary care in rural areas: improving the effectiveness of the two-step floating catchment area method. Appl Geograp. (2009) 29:533–41. doi: 10.1016/j.apgeog.2008.12.003

[33] Delamater LP. Spatial accessibility in suboptimally configured health care systems: a modified two-step floating catchment area (M2SFCA) metric. Health Place. (2013) 24:30–43. doi: 10.1016/j.healthplace.2013.07.012 24021921

[34] Wan N Zou B Sternberg T. A three-step floating catchment area method for analyzing spatial access to health services. Int J Geogr Inf Sci. (2012) 26:1073–89. doi: 10.1080/13658816.2011.624987

[35] Dony CC Delmelle ME Delmelle CE. Re-conceptualizing accessibility to parks in multi-modal cities: a variable-width floating catchment area (VFCA) method. Landsc Urban Plan. (2015) 143:90–9. doi: 10.1016/j.landurbplan.2015.06.011

[36] Hu S Song W Li C Lu J. A multi-mode Gaussian-based two-step floating catchment area method for measuring accessibility of urban parks. Cities (2020) 105:102815. doi: 10.1016/j.cities.2020.102815

[37] Xing L Liu Y Liu X. Measuring spatial disparity in accessibility with a multi-mode method based on park green spaces classification in Wuhan, China. Applied Geography (2018) 94:251–61. doi: 10.1016/j.apgeog.2018.03.014

[38] Liang H Yan Q Yan Y Zhang Q. Using an improved 3SFCA method to assess inequities associated with multimodal accessibility to green spaces based on mismatches between supply and demand in the metropolitan of Shanghai, China. Sustain Cities Soc. (2023) 91:104456. doi: 10.1016/j.scs.2023.104456

[39] Delbosc A Currie G. Using lorenz curves to assess public transport equity. J Transp Geogr. (2011) 19:1252–59. doi: 10.1016/j.jtrangeo.2011.02.008

[40] Wartenberg D. Multivariate spatial correlation: a method for exploratory geographical analysis. (1985) 17:263–83. doi: 10.1111/j.1538-4632.1985.tb00849.x

[41] Anselin Luc. Local indicators of spatial association—LISA. Geogr Anal. (1995) 27:93–115. doi: 10.1111/j.1538-4632.1995.tb00338.x

[42] Martilla AJ James CJ. Importance-performance analysis. J Mark. (1977) 41:77–9. doi: 10.1177/002224297704100112

[43] Tatem JA. WorldPop, open data for spatial demography. Scientific Data. (2017) 4:170004. doi: 10.1038/sdata.2017.4 28140397 PMC5283060

[44] Chen P Wang W Qian C . Gravity-based models for evaluating urban park accessibility: Why does localized selection of attractiveness factors and travel modes matter? Environ Plan B: Urban Anal City Sci. (2024) 51:904–22. doi: 10.1177/23998083231206168

[45] Chukwu M Huang X Wang S Li X Wei H. Urban park accessibility assessment using human mobility data: a systematic review. Annals of GIS. (2024) 30:181–98. doi: 10.1080/19475683.2024.2341700

[46] Li F Li F Li S Long Y. Deciphering the recreational use of urban parks: Experiments using multi-source big data for all Chinese cities. Sci Total Environ. (2020) 701:134896. doi: 10.1016/j.scitotenv.2019.134896 31706210

[47] Ye Y Xiang Y Qiu H Li X. Revealing urban greenspace accessibility inequity using the carrying capacity-based 3SFCA method and location big data. Sustain Cities Soc. (2024) 108:105513. doi: 10.1016/j.scs.2024.105513

[48] Deng Z Ning L. Inequity in accessibility to urban parks in environmental gentrification areas based on Multi-G3SFCA: a case study of Wuhan's main urban districts. Ecol Indic. (2024) 169:112899. doi: 10.1016/j.ecolind.2024.112899

[49] Sun Y Li H Guo X Guo C. Bridging the green space divide: a big data-driven analysis of park accessibility inequities in Chinese megacities using enhanced 3SFCA modeling. Sustainability. (2025) 17:2059. doi: 10.3390/su17052059

[50] Jin Y He R Hong J Luo D Xiong G. Assessing the accessibility and equity of Urban green spaces from supply and demand perspectives: a case study of a mountainous city in China. Land. (2023) 12:1793. doi: 10.3390/land12091793

[51] Anselin L Syabri I Kho Y. GeoDa: an introduction to spatial data analysis. *Geograpl Anal.* (2006) 38:5–22. doi: 10.1111/j.0016-7363.2005.00671.x

[52] Li D Zang H Guan Z Yu D. Study on spatial distribution and inequity of rail transit travel accessibility under multi modal traveling: a case study of Beijing. Sci Rep. (2025) 15:15823. doi: 10.1038/s41598-025-97066-4 40328882 PMC12055987

[53] Park K Rigolon A Choi D . Transit to parks: an environmental justice study of transit access to large parks in the U.S. West. Urban For Urban Green. (2021) 60:127055. doi: 10.1016/j.ufug.2021.127055

[54] Le Texier M Schiel K Caruso G. The provision of urban green space and its accessibility: Spatial data effects in Brussels. PLoS One. (2018) 13:e0204684. doi: 10.1371/journal.pone.0204684 30332449 PMC6192568

[55] Ke X Huang D Zhou T Men H. Contribution of non-park green space to the equity of urban green space accessibility. Ecol Indic. (2023) 146:109855. doi: 10.1016/j.ecolind.2022.109855

