# Supplementary material for: Spatial variations in urban park accessibility and equity under multimodal travel: a case study of Beijing
Source: Front Public Health. 2026 Oct 9;14:1953457. doi: 10.3389/fpubh.2026.1953457 (PMC13690711; doi:10.3389/fpubh.2026.1953457)
Supplement: Supplementary file 1 [file Supplementary_file_1.pdf]

Appendix A:

Table A1. Population-grid inclusion and sensitivity results.

Panel A. Competitive-demand and focal-evaluation population sets.

Zone	Populated grids	Population (million)	Focal grids	Focal population (million)	Population in <500 grids
Urban core	5,366	11.07	3,997	10.79	2.53%
Urban fringe	3,923	4.41	2,326	4.11	6.96%
Suburb	31,101	6.64	3,765	4.31	35.08%
Citywide	40,390	22.13	10,088	19.21	13.19%

Panel B. Effect of adding all positive-population grids to competitive demand while retaining the focal evaluation set.

Mode	Baseline PWA	Demand-inclusive PWA	Change	Spearman rho	IPA Jaccard	LISA Jaccard
Walking	9.724	7.844	-19.34%	0.9997	0.994	0.981
Cycling	13.471	11.306	-16.08%	0.9956	0.967	0.936
Driving	15.197	12.766	-16.00%	0.9829	0.931	0.921

Panel C. Population-weighted zonal accessibility (m2/person) under three population-grid specifications.

Scenario	Mode	Urban core	Urban fringe	Suburb	Zonal order
A: Original	Walking	5.44	12.03	18.23	Suburb > fringe > core
A: Original	Cycling	8.81	15.67	23.05	Suburb > fringe > core
A: Original	Driving	9.33	16.81	28.33	Suburb > fringe > core
B: Demand-inclusive	Walking	5.01	10.51	12.40	Suburb > fringe > core
B: Demand-inclusive	Cycling	8.17	14.15	16.43	Suburb > fringe > core
B: Demand-inclusive	Driving	8.90	15.15	20.17	Suburb > fringe > core
C: Strict all-positive	Walking	7.51	19.91	17.33	Fringe > suburb > core
C: Strict all-positive	Cycling	8.71	15.56	18.14	Suburb > fringe > core
C: Strict all-positive	Driving	9.21	16.34	21.14	Suburb > fringe > core

Notes: Scenario A uses grids with $Pop_i \geqslant 500$ for both demand and evaluation. Scenario B includes all Pop_i > 0 grids in competitive demand but retains Pop_i >= 500 grids for evaluation. Scenario C

uses all Pop_i > 0 grids for both demand and evaluation. PWA denotes population-weighted accessibility. Spearman rho is calculated for log(1 + accessibility). IPA Jaccard refers to Priority grids; LISA Jaccard refers to high-population-low-accessibility grids.

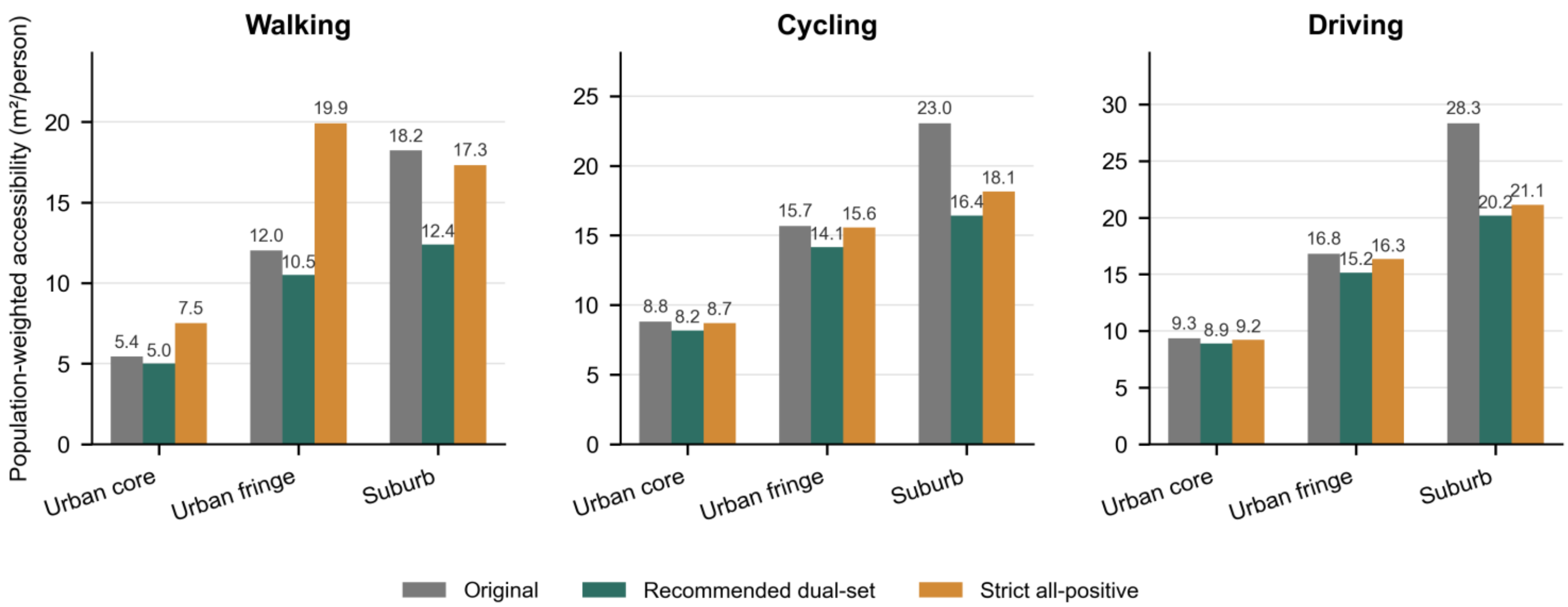

Figure A1. Population-weighted park accessibility in the urban core, urban fringe, and suburb under the original, demand-inclusive, and strict all-positive population-grid specifications. The urban core remains lowest for every mode. The walking order of the urban fringe and suburb reverses only under the strict all-positive evaluation.

This is a provisional file, not the final typeset article
